# Effects of Censoring Explicit Language in Music on Resistance Exercise Performance

**DOI:** 10.3390/jfmk10020224

**Published:** 2025-06-10

**Authors:** Christopher G. Ballmann, Sophia L. Porrill, Rebecca R. Rogers, Zachary H. Ervin, Brittany R. Neal, Haley M. Nguyen, Phoebe N. Spears, Jonathan E. Strickland, Jesus Zavala, Nicholas B. Washmuth

**Affiliations:** 1 Department of Human Studies, University of Alabama at Birmingham, Birmingham, AL 35294, USA; sporrill@uab.edu (S.L.P.); zhervin@uab.edu (Z.H.E.); brneal@uab.edu (B.R.N.); nguyenhm@uab.edu (H.M.N.); pnspears@uab.edu (P.N.S.); 2 Department of Physical Therapy, University of Alabama at Birmingham, Birmingham, AL 35294, USA; jstrick9@uab.edu (J.E.S.); zavala@uab.edu (J.Z.); 3 Department of Family and Community Medicine, University of Alabama at Birmingham, Birmingham, AL 35294, USA; rrrogers@uab.edu; 4 Department of Psychology, The University of Alabama in Huntsville, Huntsville, AL 35899, USA; nw0077@uah.edu

**Keywords:** bench press, motivation, explicit language, psychological arousal

## Abstract

**Background:** Listening to music has been widely reported to improve resistance exercise performance. However, few studies have considered lyrical content. The act of using explicit language has been shown to alter performance and psychophysiological responses to exercise. Although explicit language is widely used in mainstream music, it is unknown if altering explicit lyric content in music influences performance and psychophysiological responses to resistance exercise. Thus, the purpose of this study was to investigate the effects of censoring explicit lyrical music on bench press performance and psychophysiological responses to exercise. **Methods:** In a counterbalanced crossover manner, resistance-trained males (*n* = 11) were subjected to two conditions, namely (1) explicit music (EM) or (2) censored music (CM). Following a warm-up, music played continuously as participants completed 2 sets × 2 repetitions as explosively as possible, while a linear position transducer monitored the mean velocity of the barbell. Participants then completed 3 sets × repetitions to failure (RTFs) at 60% of a 1-repetition maximum (1-RM) separated by 2 min of rest. Motivation to exercise, psychological arousal, and rating of perceived exertion (RPE) were measured post-exercise. Total RTFs, mean velocity, motivation, psychological arousal, and RPE were compared between music conditions. **Results:** Findings show that total RTFs (*p* = 0.012) was significantly lower with CM versus EM, while mean velocity (*p* = 0.844) was not different between conditions. Psychological arousal (*p* = 0.005) and motivation (*p* = 0.002) were lower with CM versus EM. CM also resulted in a higher RPE (*p* = 0.011) compared to EM. **Conclusions:** Findings suggest that CM results in worse repetition volume compared to EM during resistance exercise but does not influence explosive ability. Changes in performance may be due to underlying decreases in motivation and psychological arousal. CM may also cause less dissociation, as evidenced by a higher RPE. Future research investigating the effects of lyrical content on exercise performance is warranted to further support current findings.

## 1. Introduction

Listening to music during exercise has been widely shown to improve performance in a variety of exercise modalities including endurance, sprint, and resistance exercise [1,2,3]. Musical compositions are multi-faceted with various components altering how the music is perceived, such as genre, tempo, volume, timbre, and lyrics. Previous evidence has suggested that modifying characteristics of music may alter exercise performance differently [4,5,6]. However, little attention has been devoted to understanding how altering the lyrical content of music influences ergogenic effects. Vocalizing explicit language has recently been shown to improve physical performance, and explicit language is commonly used in modern-day mainstream music [7,8,9]. However, it is not well understood if censoring explicit language in musical compositions influences the ergogenic effects of music during exercise.

Music has been well-described to impose ergogenic effects during bench press exercise [1,10,11,12]. Ergogenic effects have been shown in both explosive lifting and muscular endurance. Bartolomei et al. showed that self-selected music resulted in greater repetition volume at moderate loads (60% of maximal strength) during bench press exercise [10]. Furthermore, Ballmann et al. showed that pre-task music resulted in greater explosive ability through increased barbell velocity with concomitant increases in total repetitions completed [13]. These effects were likely mediated by increases in motivation and arousal. Indeed, numerous investigations have shown increases in motivation with self-selected music, which may lead to improved effort [13,14]. Furthermore, music may induce a “psyching up” effect, leading to greater arousal [15]. Collectively, music results in ergogenic effects and improved performance in bench press, although the influences of the inherent characteristics of music are still being elucidated.

The act of swearing or vocalizing explicit language has been reported to improve various aspects of exercise performance [7,16,17,18]. For example, Stephens et al. showed that using explicit language resulted in improvements in grip strength and power development during sprinting [8]. Furthermore, Jiannine et al. showed that the use of explicit language improved muscular endurance during push-ups to fatigue and extended time to exhaustion during isometric exercise [17]. While underlying mechanisms are still being clarified, improvements in performance with explicit language are thought to be due to acute psychological and physiological changes. Explicit language has been purported to result in enhanced autonomic arousal, which may then lead to greater effort or decreased nociception [7,19]. Furthermore, other investigations have shown improvements in mood following exercise when explicit language was repeated during effort. Overall, explicit language has been suggested to improve physical performance, but few investigations have delineated if these effects are present with explicit language in music.

To date, few studies have investigated how lyrical content alters exercise performance. Zareian et al. showed that listening to lyrical music during a running test resulted in lower ratings of perceived exertion (RPE) when compared to the same song without lyrics [20]. Furthermore, Sanchez et al. showed music containing lyrics results in faster pedal cadence during cycling compared to no lyrics, although the RPE and affect remained unchanged [21]. However, neither of these investigations accounted for lyrical content and rather focused on the presence of lyrics and their effects on exercise performance. Given the recent findings of improvements in performance with vocalizing explicit language and the high prevalence of explicit language in modern-day music, understanding the role of explicit lyrical content in music on potential ergogenic effects is especially relevant. Therefore, the purpose of this study was to investigate the effects of censoring explicit lyrical music on bench press performance and psychophysiological responses to exercise. We hypothesized that music with explicit lyrics would result in superior performance and psychophysiological responses to exercise compared to music that was censored.

## 2. Materials and Methods

### 2.1. Study Design

The following study utilized a randomized crossover counterbalanced design. Participants were randomized into two experimental music conditions, involving (1) explicit music (EM) and (2) censored music (CM). Participants completed a one-repetition maximum (1-RM) test for bench press on the first visit. In the subsequent 2 experimental visits, participants completed explosive bench press exercises to detect barbell velocity, followed by muscular endurance testing of three sets of repetitions to failure (RTFs) while listening to the corresponding music condition. Motivation, rating of perceived exertion (RPE), and psychological arousal were documented after exercise. Comparisons were drawn between music conditions, and all study visits were separated by 48 h to allow for recovery.

### 2.2. Participants

To determine an adequate sample size, an a priori analysis was conducted using G-power 3.1.9.6 open access software [22]. A previous investigation from our lab showed improvements in total repetition volume with music listening with an estimated effect size of d = 0.985 [13]. Therefore, the following parameters were used: test = matched pairs *t*-test, d = 0.985, α = 0.05, β = 0.8. Adequate sample size was calculated to be *n* = 11. Accordingly, resistance-trained males (*n* = 11) were recruited to participate. Descriptive characteristics are shown in Table 1. Inclusion criteria included engaging in resistance training 2–3 days/week [23], with no musculoskeletal injuries in the past 6 months limited to training, and no chronic disease. To ensure the safety of exercise participation, participants were screened using a physical activity readiness questionnaire [23]. Before each visit, participants were asked to refrain from caffeine, nicotine, and alcohol 12 h prior and vigorous upper body exercise 24 h prior [23]. Participants were also asked to maintain similar dietary and sleep habits prior to each visit. Verbal and written informed consent were obtained from each participant, and all experimental procedures were conducted in accordance with the Declaration of Helsinki and approved by the University of Alabama at the Birmingham Institutional Review Board (IRB) (UAB IRB-300012824; 19 November 2024).

### 2.3. One-Repetition Maximum (1-RM) and Familiarization

On the first visit for each participant, one-repetition maximum (1-RM) for bench press was determined, and participants were familiarized with lifting with maximum explosiveness (for velocity testing), as previously described by Ballmann et al. [13,14]. Participants warmed up with 5 repetitions of 40% followed by 3 repetitions of 60% of self-reported 1-RM, and each set was separated by 3 min of rest. Following this, the weight of the barbell was progressively increased by 2.5–20.0 kg for a single attempt until the concentric phase of the lift could not be completed [23]. Participants rested for 5 min between each 1-RM attempt. To familiarize participants with lifting explosively, a standard 20 kg Olympic bar was lifted as fast and explosively as possible during the concentric phase. Form was corrected and demonstrated as needed. This was also reinforced during the warm-up sets for subsequent visits. The 1-RM obtained was used to determine the load percentage for experimental visits.

### 2.4. Explicit and Censored Music

Following the 1-RM and familiarization visit, participants were provided with a pre-determined list of 30 songs containing explicit lyrics from 5 different genres, including rock n’ roll, pop, rap, rhythm and blues, and electronic dance music. Participants were instructed to self-select a single song from the playlist, which was used for both experimental conditions. The songs chosen had an average of 29 explicit words ± 4 and an average tempo of 137 bpm ± 8. For the EM condition, all lyrics, including explicit lyrics, were left intact. For the CM condition, publicly available song versions were used that were censored according to the U.S. Federal Communications Commission guidelines on the Broadcast of Obscenity, Indecency, and Profanity [24]. Explicit lyrics were removed such that the absence of the lyric resulted in a “muting” of language solely for the word of interest. Music was played through Bluetooth headphones (Apple Inc., Cupertino, CA, USA) using a single electronic device. All songs had a minimum medium tempo of 120 bpm [25,26]. Volume was standardized to the same level for all participants for each experimental visit to avoid potential confounding effects [27].

### 2.5. Procedures

During each experimental visit, participants completed a battery of bench press tests while listening to corresponding music conditions similarly, as previously described by Williams et al. [28]. Participants began by completing a standardized bench press warm-up of 5 repetitions at 40% 1-RM followed by 5 repetitions at 50% 1-RM. Each set was separated by 3 min of rest. Following the warm-up, music began playing and was continuously played on repeat for the entirety of the bench press exercise protocol. To assess explosive ability, participants then completed 2 sets × 2 repetitions at 60% 1-RM as explosively as possible separated by 2 min of rest. During this, barbell velocity was detected using a linear position transducer (GymAware; Kinetitech Performance Technology, Australian Capitol Territory, Australia). This device has been previously validated for measuring velocity during barbell resistance exercise [29,30]. The highest velocity of the 2-rep average between sets was used for analysis. Following another 2 min rest period, muscular endurance was assessed by having participants complete 3 sets × repetitions to failure (RTF) at 60% 1-RM separated by 2 min of rest. Total and set repetition volumes were collected for analysis. Following exercise, motivation and psychological arousal were measured using visual analog scales as previously reported [15,31,32,33]. Briefly, participants were instructed to mark on a 100 mm line how they felt during exercise where 0 was the absence of feeling motivated/psyched up and 100 was feeling extremely motivated/psyched up. A rating of perceived exertion (RPE) was also documented post-exercise using a 1–10 scale.

### 2.6. Data Analysis

All data were analyzed using Jamovi software (Version 0.9) [34,35]. Data normality was confirmed using the Shapiro–Wilk method. To determine differences in repetition volume, a 2 × 3 [condition × set] repeated-measures ANOVA was used to detect set-to-set differences. For significant main effects and interactions, a Tukey post hoc analysis was used for pairwise comparisons. Estimates of effect size for main effects and interactions were calculated using eta squared (η^2^). To determine differences in mean velocity, motivation, psychological arousal, and RPE, a paired samples *t*-test was used. Cohen’s d effect sizes were used for estimates of effect sizes for *t*-tests [36,37]. All data are presented as mean ± standard deviation (SD). Significance was set at *p* ≤ 0.05.

## 3. Results

### 3.1. Repetitions and Mean Velocity

**Figure 1:**
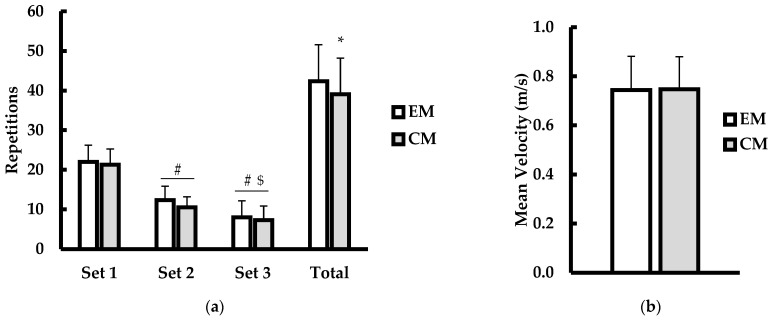
(**a**) Set-to-set and total repetitions; (**b**) mean velocity (m·s^−1^) between explicit (EM; white bars) and censored (CM; gray bars) music conditions. Data are presented as mean ± SD. # indicates significantly different from Set 1 (*p* ≤ 0.05). $ indicates significantly different from Set 2 (*p* ≤ 0.05). * indicates significantly different from EM (*p* ≤ 0.05).

Repetitions to failure (reps) are shown in Figure 1a. There was a significant main effect for condition (*p* = 0.012; η^2^ = 0.006) and set (*p* < 0.001; η^2^ = 0.729) but no interaction for condition × set (*p* = 0.321; η^2^ = 0.001). Post hoc analysis for condition showed that CM resulted in a lower number of repetitions completed compared to EM (*p* = 0.012; d = 0.929). For set, participants completed a greater number of repetitions during the first set compared to the second (*p* < 0.001; d = 4.2) and third (*p* < 0.001; d = 3.79) sets regardless of condition. More repetitions were completed during the second set (*p* = 0.001; d = 1.5) compared to the third set, regardless of condition. Mean velocity (m·s^−1^) is shown in Figure 1b. There were no significant differences in mean velocity between EM and CM conditions (*p* = 0.844; d = 0.06).

### 3.2. Rating of Perceived Exertion, Motivation, and Arousal

**Figure 2:**
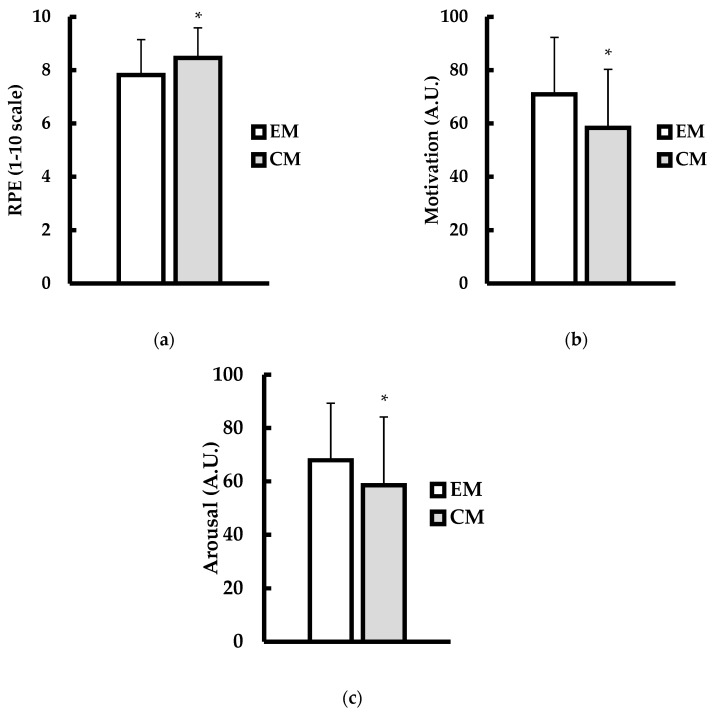
(**a**) Rating of perceived exertion (RPE; 1–10 scale), (**b**) motivation (arbitrary units; A.U.), and (**c**) arousal (arbitrary units; A.U.) between explicit (EM; white bars) and censored (CM; gray bars) music conditions. Data are presented as mean ± SD. * indicates significantly different from EM (*p* ≤ 0.05).

The rating of perceived exertion (1–10 scale; RPE) is shown in Figure 2a. Analysis revealed that RPE with CM was significantly higher than EM (*p* = 0.011; d = 0.94). Motivation (arbitrary units; A.U.) is shown in Figure 2b. Motivation levels were significantly lower with CM versus EM (*p* = 0.002; d = 1.2). Psychological arousal (arbitrary units; A.U.) is shown in Figure 2c. Listening to CM resulted in significantly lower arousal compared to EM (*p* = 0.005; d = 1.01).

## 4. Discussion

While music has been widely reported to possess ergogenic effects, little attention has been devoted to how lyrical content may affect performance. Recently, verbalizing explicit language has been shown to improve physical performance, including muscular endurance [7,17]. However, it remains unclear if explicit language in music alters the ergogenic potential of music. Thus, this study sought to elucidate whether censoring explicit language in music alters bench press performance and psychophysiological responses to resistance exercise. Findings reveal for the first time that censoring explicit language in self-selected music resulted in poorer muscular endurance, as evidenced by less repetition volume accumulated over repeated bench press exercises. Furthermore, CM resulted in a worsening of psychophysiological responses to exercise, including lower motivation, arousal, and higher levels of RPE. These results may provide novel evidence for athletes and practitioners, informing the optimization of music to enhance performance.

Although no other investigations on censored music and exercise performance exist, current findings of worsened repetition volume with censored music support previous findings of vocalizing explicit language. Jiannine et al. reported a 15% decrease in the number of pushups to fatigue when repeating neutral language versus explicit language [17]. Furthermore, muscular endurance during isometric exercise during wall sits and planks was 22% and 12% worse with neutral versus explicit language, respectively. When compared with current findings, this suggests that explicit language influences muscular endurance whether spoken or listened to in music. While mechanisms are not fully clear from current data alone, worsening in performance may be due to maladaptive changes in psychophysiological outcomes such as motivation and RPE. Indeed, increases in motivation from music may lead to greater effort and thus improve performance, which was less apparent with censoring music [1,13,14,15]. Furthermore, dissociation from discomfort with music may lead to improved exercise volume attained [38,39,40]. Since the current findings showed increases in RPE, participants may not have been able to dissociate, thereby increasing feelings of fatigue during the repeated bench press exercise. Interestingly, explosive performance, as assessed through the mean velocity of barbell movement, remained unchanged regardless of lyric censoring. This was counter to the primary hypothesis and is in opposition to previous music studies, albeit without considering lyrical content [1,13]. The reasons for these disparities are not fully clear but may be due to the short nature of the explosive bench press testing. It is plausible that since the velocity testing was non-fatiguing exercise, the censoring of music had little impact. Since one of the apparent mechanisms for censoring music from the current dataset was increasing RPE, this likely had little impact on non-fatiguing exercise during explosive lifts. However, further investigation of whether censoring music influences explosive ability during fatiguing exercise is needed to confirm this.

Censoring music led to profound alterations in psychophysiological outcomes, with the current findings suggesting motivation, perceptions of exercise intensity, and arousal were negatively impacted. While no evidence exists to explain this directly in the context of censored music, investigations in verbalizing explicit language may allow for speculation. Explicit language has been linked to altered emotional responses, which likely lead to increased arousal and motivation, such as that in the current study [41]. This may be reflective of the activation of areas in the brain responsible for motor and emotional processing (e.g., amygdala), which have been linked to heightened arousal and physical performance [7,42,43]. Furthermore, music has been shown to increase amygdala activation and may impact motivational behavior [44]. This is further bolstered by numerous investigations showing increases in perceptions of motivation and arousal with music similar to that currently measured [1,13,14]. It should be noted that the previously mentioned mechanism was not established currently and thus should be interpreted with caution as a hypothetical possibility. Furthermore, the removal of explicit language may have led to less dissociation, as evidenced by higher RPE levels, since previous evidence of verbalizing explicit language has suggested a greater dissociation effect [7,45]. In direct comparison to previous music research, there is a myriad of evidence showing that preference mediates the psychophysiological responses to exercise [1]. While not directly measured, it is plausible that censoring music was less preferred by participants and thereby caused poorer responses. This would explain the lower motivation, arousal, and higher RPE, as these responses align with those of previous investigations when participants listened to non-preferred music [13,14,15]. However, the preference for explicit language was not currently measured, and more research delineating the individual preference for explicit language is warranted.

While the current study is the first to investigate the effects of censoring explicit lyrics in music on exercise performance, there were several limitations. First, there was not a no-music control included in the experiments and, thus, findings can only apply knowledge for explicit versus censored music and cannot conclude as to whether censored music is superior to no music. However, it should be mentioned that self-selected music versus no music or non-preferred music has been well documented to result in superior performance [1,3,10,13,41]. Also, all psychophysiological measures were subjective and as such may be prone to more participant bias than objective measures [46]. However, large effect sizes currently seen suggest a robust and consistent response. Not all songs had the same number of explicit lyrics, and only a select number of genres were chosen by participants. As such, the stimulus may have been different from participant to participant and may vary for individuals who listen to other genres. While participants were allowed to self-select their music, which was paramount in controlling for song preference, the preference for explicit language was not accounted for, and their removal may have also impacted expectations. Therefore, current results may not apply to all music listeners or songs, and future investigations should elucidate the possibility of different responses based on explicit language preference. Lastly, the current sample consisted of young resistance-trained males from a small geographical area. Therefore, current findings may not be generalizable to other groups of different biological sex, training status, age, and cultural background with varying language sensitivity.

## 5. Conclusions

In conclusion, censoring explicit language in music results in diminished muscular endurance during bench press exercises and negatively alters motivation, arousal, and RPEs. However, barbell velocity remained unchanged. From a practical standpoint, explicit language in modern music is used abundantly, especially in certain genres. Present findings suggest that censoring music may compromise performance and psychophysiological responses to exercise training. Thus, athletes and coaches who employ music as an ergogenic tool may want to avoid censoring music to optimize performance, especially during fatiguing exercises where muscular endurance is of importance.

## Figures and Tables

**Table 1:** Descriptive characteristics of participants.

Descriptive Characteristics (*n* = 11)	Mean ± SD
Age (years)	23.9 ± 3.8
Height (cm)	175.2 ± 6.2
Body Mass (kg)	83.9 ± 12.6
RT Experiences (years)	7.6 ± 2.9
1-RM (kg)	122.5 ± 15.7
Relative 1-RM (kg)	1.5 ± 0.4

Resistance training (RT); one-repetition maximum (1-RM); relative 1-RM = 1-RM/body mass.

## Data Availability

The original contributions presented in this study are included in the article. Further inquiries can be directed to the corresponding author.
